# Filter feeding, deviations from bilateral symmetry, developmental noise, and heterochrony of hemichordate and cephalochordate gills

**DOI:** 10.1002/ece3.6962

**Published:** 2020-11-03

**Authors:** Charles Larouche‐Bilodeau, Xavier Guilbeault‐Mayers, Christopher B. Cameron

**Affiliations:** ^1^ Sciences biologiques Université de Montréal Montreal QC Canada

**Keywords:** Cephalochordata, Deuterostomia, ectopic expression, filter feeding, fluctuating asymmetry, gill slits, Hemichordata

## Abstract

We measured gill slit fluctuating asymmetry (FA), a measure of developmental noise, in adults of three invertebrate deuterostomes with different feeding modes: the cephalochordate *Branchiostoma floridae* (an obligate filter feeder), the enteropneusts *Protoglossus graveolens* (a facultative filter feeder/deposit feeder) and *Saccoglossus bromophenolosus* (a deposit feeder). FA was substantially and significantly low in *B. floridae* and *P. graveolens* and high in *S. bromophenolosus*. Our results suggest that the gills of species that have experienced a relaxation of the filter feeding trait exhibit elevated FA. We found that the timing of development of the secondary collagenous gill bars, compared to the primary gill bars, was highly variable in *P. graveolens* but not the other two species, demonstrating an independence of gill FA from gill bar heterochrony. We also discovered the occasional ectopic expression of a second set of paired gills posterior to the first set of gills in the enteropneusts and that these were more common in *S. bromophenolosus*. Moreover, our finding that gill slits in enteropneusts exhibit bilateral symmetry suggests that the left‐sidedness of larval cephalochordate gills, and the directional asymmetry of Cambrian stylophoran echinoderm fossil gills, evolved independently from a bilaterally symmetrical ancestor.

## Graphical abstract

We measured gill slit fluctuating asymmetry (FA) and developmental stability, in three species of invertebrate deuterostomes with different feeding modes: the cephalochordate *Branchiostoma floridae* (an obligate filter feeder), and the enteropneusts *Protoglossus graveolens* (a facultative filter feeder/deposit feeder) and *Saccoglossus bromophenolosus* (a deposit feeder). Our results suggest that the gills of species that have experienced a relaxation of the filter feeding trait exhibit elevated FA and developmental noise.

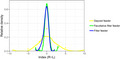

## 1 INTRODUCTION

In bilaterian organisms, structures can be symmetrical or asymmetrical, the latter being subdivided into three forms: antisymmetry, directional asymmetry, and fluctuating asymmetry (Endler, 1986; Lahti et al., 2009). Fluctuating asymmetry (FA) consists of random deviations from perfect bilateral symmetry on a population of organism (Graham et al., 2010). This variation around the perfect symmetrical distribution represents a measure of development noise or developmental instability (Rott, 2003). As both sides of a bilateral trait develop under the control of the same genome, the developmental phenotypic target of a population should be perfect symmetry (De Coster et al., 2013), but developmental noise results in deviations from perfect bilateral symmetry or an increase in FA (Emlen et al., 1993; Møller & Swaddle, 1997).

A high level of fluctuating asymmetry and high variability in size and number of a phenotype are signs of reduced functionality and relaxed selective pressure where drift predominates. This is conspicuous when homologous structures are compared that show high functionality versus a relaxation in that function (Guthrie, 1965; Tague, 1997). In those cases where function is lost, the relaxed selective pressure may result in many paths or scenarios (Lahti et al., 2009). A trait that has lost its function may be lost, become vestigial, persist, or experience exaptation. Vestigialization is expected when the trait bears a cost without benefits and may lead to the loss of the trait. Persistence is when a trait remains, when the relation between cost and benefits is the other way around or null (Lahti et al., 2009). Exaptation is when a phenotypic trait remains for a function than is different than its original role (Dorken et al., 2004; Lahti et al., 2009; Tague, 1997).

**Figure 1:**
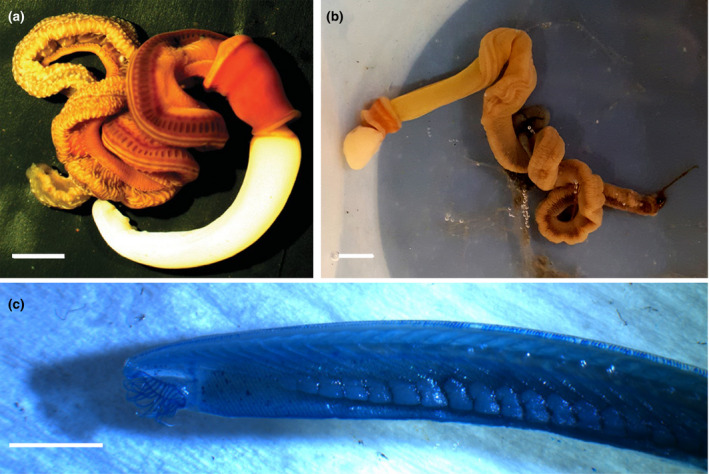
Photographs of (a) *Saccoglossus bromophenolosus*, (b) *Protoglossus graveolens,* and (c) *Branchiostoma floridae*. (a) and (b) were live specimens. (c) was stained and cleared. Scale bars equal 4 mm

Here, we investigate the interspecific gill symmetry and intraspecific gill fluctuating asymmetry in the cephalochordate *Branchiostoma floridae* Hubbs, 1922, and two enteropneusts; *Protoglossus graveolens* Giray & King (1996) and *Saccoglossus bromophenolosus* King et al. (1994) (Figure 1). These species are ideal to investigate gill fluctuating asymmetry for the following reasons. *B. floridae* is an obligate filter feeder (Ruppert et al., 2000), *P. graveolens* is a facultative filter feeder (Gonzalez & Cameron, 2009), and *S. bromophenolosus* is a deposit feeder that does not filter food particulates (Cameron, 2016; Knight‐Jones, 1953), which allows us to relate gill function to gill fluctuating asymmetry. Gills are a discrete, rather than a continuous trait and therefore less prone to measurement error (Palmer & Strobeck, 1986). The three species are in the same adult size range, and their gills show high repeatability. These gills show no evidence of wear that can complicate left‐right comparisons (Rott, 2003), and finally, the acorn worms live in the same habitat, so differences in gill symmetry are not due to the environment.

**Figure 2:**
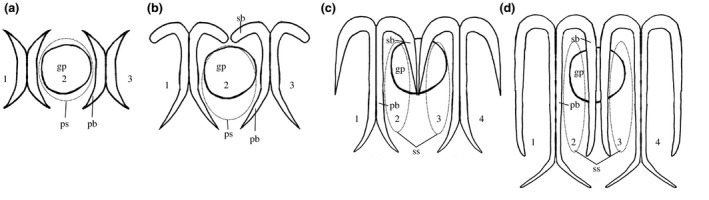
Line drawing of the developmental stages of the gill slits and bars. Note that gill pores are ectodermal and drawn as solid lines, whereas gill slits and bars are endodermal and the slits are drawn as dotted lines. (a) Early gill slits are round in circumference and bordered by small lateral primary gill bars. (b) The primary gill bars develop around the gill slit elongating it to form the early stage secondary gill bars. (c) The secondary gill bars elongate ventrally, dividing the primary gill slit into two elongated secondary gill slits. (d) The secondary gill bars are fully developed. Gill slits are numbered in each drawing representing how we counted them gp, gill pore; pb, primary gill bar; ps, primary gill slit; sb, secondary gill bar; ss, secondary gill slit

Gills are a shared, plesiomorphic feature of the deuterostomes. This homology has been established from comparative morphology, molecular development, comparative genomics, and the fossil record. Hemichordate and cephalochordate gills are dorsolaterally located, frequently paired, and connect the inner pharyngeal cavity to the environment (Cameron, 2002; Gonzalez & Cameron, 2009; Ruppert et al., 2000). Gills begin development as a simple pore, which is then elongated dorsal to ventral by extension of collagenous gill bars, creating a primary gill slit. This slit is then divided into two secondary gill slits by the downward projection of a secondary gill bar (or tongue bar) resulting in a series of M‐shaped collagenous gill bars. As worms grow, gills are added posteriorly first as pores, then as primary gill slits bordered by primary gill bars, and finally as secondary gill slits when the primary gill slit is divided by the downward growth of secondary gill bars (Figure 2). The gill bars are endodermal collagenous (type II) skeleton (Cameron, 2005; Ogasawara et al., 1999; Philippe et al., 2011; Röttinger & Lowe, 2012; Rychel et al., 2006; Rychel & Swalla, 2007; Sato & Holland, 2008; Satoh, 2008). Genes expressed during the development of gills of hemichordates and chordates include Pax1/9, Nkx2.1, Nkx2.2, Fox A, FoxC, FoxL1, FoxI, Eya, and Six1 (Fritzenwanker et al., 2014; Gerhart et al., 2005; Gillis et al., 2012; Ogasawara et al., 1999; Okai et al., 2000). The first four genes are transcription factors that are arranged in a synteny unique to the deuterostomes, including echinoderms (Simakov et al., 2015).

The symmetry of the ancestral deuterostome is a subject of debate because living and fossil species may be bilateral or directionally asymmetrical (Cameron, 2016; Jefferies, 2001; Sato & Holland, 2008; Zamora & Rahman, 2014). Deuterostomes are comprised of two major branches, and the acorn worms are thought to most closely resemble the ancestral Ambulacraria (including echinoderms) whereas the cephalochordates the ancestral Chordata (including tunicates and vertebrates). Gills of Cambrian fossil (Caron et al., 2013; Nanglu et al., 2016) and living acorn worms have not been quantified but are assumed to be symmetrical. Gills of stem echinoderms (Jefferies, 2001; Jefferies et al., 1996; Rahman et al., 2015; Smith, 2008; Smith et al., 2013; Zamora & Rahman, 2014; Zamora et al., 2012) and larval cephalochordates (Boorman & Shimeld, 2002; Dominguez et al., 2002; Willey, 1891) exhibit directional asymmetry. Here, we quantify the symmetry of the gills of an adult cephalochordate and two acorn worms. We also count the number of posterior gill slit pairs that have primary gill bars but lack secondary gill bars, as a measure of heterochrony. Finally, we show that some acorn worms develop a second gill complex, which we interpret as a previously unknown form of gill developmental error.

## 2 MATERIALS AND METHODS

### 2.1 Specimen collection and treatment

The enteropneust *Saccoglossus bromophenolosus* (*n* = 65) and *Protoglossus graveolens* (*n* = 50) were dug at low tide, near to the old brickyard in Lowe’s Cove, adjacent to the Darling Marine Center, University of Maine. The enteropneusts live in sediment composed of clay, mixed with organic calcium carbonate debris and sand. These two acorn worm species are sympatric and often collected from the same shovel of sediment, and thus, differences in their FA are more likely due to differences in function rather than the environment. The cephalochordate *Branchiostoma floridae* (*n* = 50) were collected in Tampa Bay in the 1980s, fixed in formalin, and stored in 70% alcohol in the department of biological sciences, Université de Montréal. Adapting the methods from Inouye (1976) and Dingerkus and Uhler (1977), the acorn worm specimens were relaxed in a 7% solution of magnesium chloride and fixed in a 10% formalin solution. All specimens were then dehydrated in a sequential series of ethanol (i.e., 30%, 50%, 70%, 95%, and 99%) for an hour at each step. Subsequently, they were stained in a solution of 0.1 % Alcian blue 8GX, 5% acetic acid, and 70% ethanol for 24 hr, neutralized in a 1% solution of potassium hydroxide for a further 24 hr, cleared with a 1% pancreatic trypsin buffer solution, dissected, and then the left and right gill slits were counted.

### 2.2 Data collection

We counted only fully developed gill slits bordered by clear gill bars, either primary or secondary. We avoided counting pores because in the early stages, they are small, lack gill bars (and Alcian stain) and so difficult to quantify with confidence. We also avoided counting very early stages of gill bar development, when they can be confused with folds in the epithelium, where Alcian blue was sometimes found. Special attention was given to the posterior gill slits. From posterior to anterior, the gills are in the earliest to latest stages of development. We counted the number of undivided primary gill slits that were bordered by primary gill bars, but not divided into secondary gill slits by the downward extension of secondary gill bars. The difference in relative number, or timing, of the primary slits compared to the secondary gill slits allowed us to compare gill slit developmental heterochrony between these three organisms. Finally, we noted a few exceptional cases where a second gill complex developed in the intestine of some acorn worms. These gills were not counted as part of our other analysis.

### 2.3 Statistical analysis

Fluctuating asymmetry of gill slits were assessed by calculating a symmetry index (R‐L) and generating a relative density of this index by species, where relative density was log transform to avoid negative values and retro‐transform for visualization. To assess departure from a perfect symmetrical distribution, four modes of distribution were calculated: the mean, the standard deviation, the skewness and the kurtosis using the function mean() and sd() from the package “stat” (R Core Team, 2018), and the function kurtosis() and skewness() from the package “moments” (Komsta & Novomestky, 2015). Subsequently, the kurtosis and skewness were compared to the 5% and 95% quantile of the distribution of those mode calculated from 100,000 rounded normal distributions (*µ* = −0.01, *σ* = 1.50). The average mean and standard deviation of the distribution of the symmetry index were used to generate the normal distributions to account for the nature of our data. As kurtosis is independent of the variance of the distribution, it served to quantify the movement of mass toward the tail of the distribution (Westfall, 2014) and the skewness served as a measure of a horizontal stretch of the distribution. Due to the lack of replication of the symmetry index (R‐L) dispersion descriptors (i.e., kurtosis and standard deviation), linear model (lm) from the package “stats” and generalized least squares (gls) from the package “nlme” (Pinheiro et al., 2018; R Core Team, 2018) were used to perform a direct assessment of the differential level of FA among species by comparing the squared residuals of the symmetry index. Linear and generalized least squares model were also used to assess difference between the overall number of gill slits among species. Variance analysis of the symmetry index was not carried out to evaluate differences in the symmetry aspect of the gill pores, as the putative absence of differences among species would be an artifact of the greater variance observed in *S. bromophenolosus* and would not be informative regarding differences in symmetry among species. Considering the experimental design of our study and the structure of the data, a Student's *t* test by permutation (*n* = 9,999) for paired sample, function t.paired.perm() (Legendre & Legendre, 2012), were used to assess the difference between both sides of the bilateral trait for each species. For all linear models, the assumptions of homogeneity of variance and normally distributed residuals were ascertained by visual inspection. The models were modified as needed with an appropriate variance function to effectively deal with heteroscedasticity, when present (Zuur et al., 2009). If the residual structure showed little difference between several models, the selection was made by comparing the AICs. In figures, letters used to denote differences among groups were based on Tukey’s HSD and were generated using the function emmeans() and CLD() from the “emmeans” packages (Lenth, 2019).

Heterochrony in the sequential development of the gill slits was assessed by calculating the mean and the standard deviation of the number of gills lacking secondary gill bars for each species.

## 3 RESULT

### 3.1 Variance and fluctuating asymmetry

**Figure 3:**
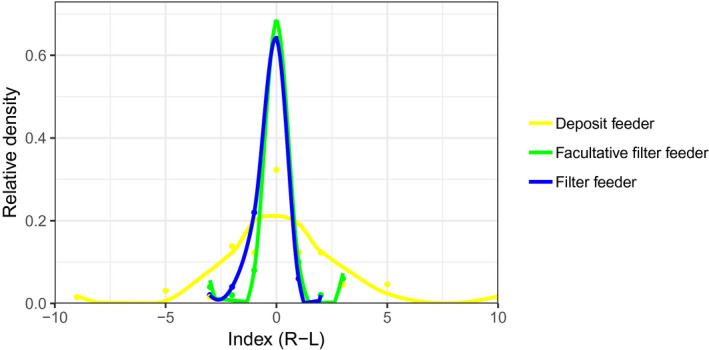
Graphical representation of the distribution of the symmetry index (R‐L) for each species. *Saccoglossus bromophenolosus* (Hemichordata) is a deposit feeder, *Protoglossus graveolens* (Hemichordata) is a facultative filter feeder, and *Branchiostoma floridae* (Cephalochordata) is an obligate filter feeder. *S. bromophenolosus*, *P. graveolens*, and *B. floridae* have symmetry index distribution means of 0.15, 0.08, and −0.26 and symmetry index distribution standard deviations of 2.57, 1.12, and 0.80, respectively

**Figure 4:**
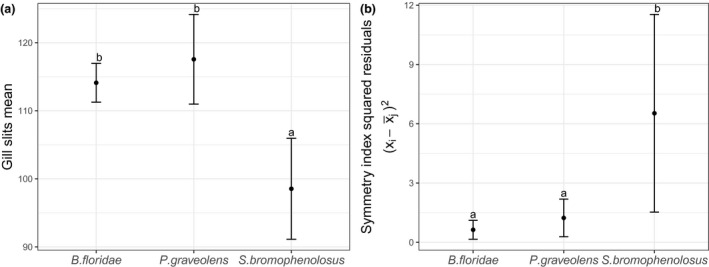
Differences (a) in number of gill slits and (b) symmetry index (R‐L) squared residuals among species. Error bars represent the 95% confidence intervals; letters above each mean represent Tukey’s honest significant difference (HSD) groupings (*p* ≤ .05)

**Figure 5:**
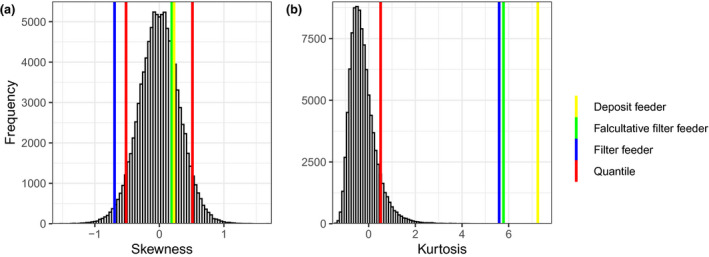
Representation of the two modes of the distribution of the symmetry index (R‐L) for each species (a) in the skewness, and (b) the kurtosis. *S. bromophenolosus*, *P. graveolens*, and *B. floridae* have a kurtosis of 7.25, 5.78, and 5.59, and a skewness of 0.23, 0.19, and −0.69, respectively. The distribution of the skewness and the kurtosis was calculated from 10,000 rounded normal distribution (*µ* = −0.01, *σ* = 1.50). In panel (a), the vertical lines beginning from the left represent the 5% quantile and the 95% quantile, respectively, and in panel (b), the vertical line represents the 95% quantile. The deposit feeder is *S. bromophenolosus,* the facultative filter feeder is *P. graveolens*, and the obligate filter feeder is the cephalochordate *B. floridae*

The acorn worms *Saccoglossus bromophenolosus* (*N* = 65), *Protoglossus graveolens* (*N* = 50), and the cephalochordate *Branchiostoma floridae* (*N* = 50) gill slits are bilaterally symmetrical and exhibit fluctuating asymmetry (Figure 3; Table 1). The standard deviation of gill pair number is greater in *Saccoglossus,* a genus that does not use the gills for filter feeding (Table 1). Similarly, the differences in symmetry index (R‐L) squared residuals among species show that only *Saccoglossus* has a significantly higher FA (*S. bromophenolus – P. graveolens*: *p* < .05, *S. bromophenolus – B. floridae*: *p* < .05 and *P. graveolens – B. floridae*: *p *> .05; Figure 4b). The number of gill slits of *S. bromophenolosus* was significantly less than *P. graveolens* and *B. floridae* (Figure 4a; *p* < .0001), and no difference was detected between the latter two species (Figure 4a; *p* > .05). Similar to the standard deviation, the kurtosis decreased as the functionality of the gill slits increased; the highest kurtosis was the deposit feeder *S. bromophenolosus*, then the facultative filter feeder *P. graveolens*, and the lowest was the obligate filter feeding cephalochordate *B. floridae* (Figure 5; Table 1). High kurtosis reveals a movement of mass toward the tail of the distribution and, therefore, an increase of FA (Figure 3). The greatest difference of left vs. right number of gills in *S. bromophenolosus* was ten, whereas a maximum of three was observed in the other species.

**Table 1:** Four of the distribution modes, squared residuals of the symmetry index (R‐L) and *p*‐value of the paired Student *t* test for each species

	Species	*S. bromophenolosus*	*P. graveolens*	*B. floridae*
	Nutritional behavior	Deposit feeder	Falcultative filter feeder	Filter feeder
Standard deviation	Symmetry index	2.57	1.12	0.80
	Right and left gill slits	24.41	18.84	8.16
Squared residuals	Symmetry index	1.68^b^	0.61^a^	0.57^a^
Mean	Symmetry index	0.15	0.08	−0.26
	Right and left gill slits	98.54^a^	117.56^b^	114.11^b^
Third mode	Kurtosis	7.25	5.78	5.59
Fourth mode	Skewness	0.23	0.19	−0.69
Paired Student t test	P‐values	0.67	0.71	0.04

Letters above each mean represent Tukey’s honestly significant difference (HSD) groupings (*p* ≤ .05).

### 3.2 Asymmetrical aspect of fluctuating asymmetry

Each of the three species exhibited fluctuating asymmetry. No left or right biases were detected for *P. graveolens* or *S. bromophenolosus* (*p *> .05), and a significant left bias (i.e., more gills were on the left side in 14 of the 18 asymmetrical individuals) was detected in the gill slits of *B. floridae* (paired Student *t* test: *p* = .04). This latter finding did not demonstrate directional asymmetry (DA) because most specimens were perfectly symmetrical (32 out of 50) and asymmetries were small deviations (up to 3 gill slits) on both sides of the symmetrical state. Skewness confirms these results: The skewness of the symmetry index of *B. floridae* was higher than expected if it was part of a perfectly symmetrical distribution (Figure 5). The paired Student's t test and the skewness of the symmetry index of *S. bromophenolosus* and *P. graveolens* were within what was expected for a symmetrical distribution (Table 1; Figure 5).

### 3.3 Sequential development of the gill slit complex

**Figure 6:**
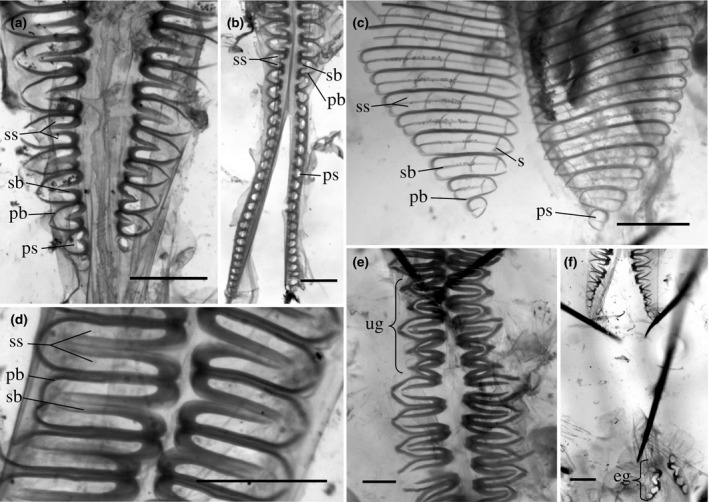
Photographs of the gill slit complexes of (a) *Saccoglossus bromophenolosus*, (b) *Protoglossus graveolens,* and (c) *Branchiostoma floridae*. (d) *S. bromophenolosus* gill slits complex in higher magnification, and (e) underdeveloped secondary gill slits following damage, and (f) a second ectopic gill complex posterior to the normal complex. Anterior is at the top with a section of cleared intestine in between. All scale bars equal 500 µm except for (b) were the scale bars equal 1,000 µm. In all photograph, the pharynx midline is dorsal except (c) where it is ventral. eg, ectopic gills; pb, primary gill bar; ps, primary gill slit; s, synapticula; sb, secondary gill bar; ss, secondary gill slit; ug, underdeveloped gill bar

New gills are added sequentially to the posterior pharynx. The posterior pairs, then, are the youngest and least developed. They begin as simple pores, which are elongated into slits by the downward growth of the primary gill bars. Each slit is then divided into two by the downward projection of the secondary gill bars (Figure 6a, b, c, & d). We found one exception to this developmental sequence. A single *S. bromophenolosus* specimen had a gill slit with a short series of underdeveloped secondary gill slits and bars between fully developed gills (Figure 6e). The number of gills that lacked secondary gill bars in the posterior pairs of gills varied between species, and thus, the comparison of absence to presence of secondary gill bars is a measure of heterochrony. The number of gills that lacked secondary gill bars was similar in *S. bromophenolosus* and *B. floridae* with an average 1 ± 0.6 (*N* = 65) and 0.6 ± 0.5 (*N* = 50), respectively, on each side (Figure 6a & c), whereas up to 29 pairs of gills lacked secondary gill bars in *P. graveolens* with a mean of 17 ± 5 (*N* = 50; Figure 6b).

### 3.4 Ectopic expression of a second gill complex in Enteropneusta

An unexpected finding of this study was that 8 of the 65 *S. bromophenolosus* specimens dissected (or 12.3%) had an additional pharyngeal complex (Figure 6f) located posterior to the end of the normal complex and separated by a length of intestine. It was typical in that it consisted of paired gill slits bordered by gill bars, and the posterior gills were less developed than the anterior gills (Figure 6f). The secondary gill bars were never fully developed. By comparison, an additional pharyngeal complex was found in one of the 50 *P. graveolens* specimens. A second ectopic gill complex and higher FA in *S. bromophenolosus* are both examples of developmental error.

## 4 DISCUSSION

Fluctuating asymmetry of the pharyngeal gill is relatively low in the obligate filter feeder *Branchiostoma floridae* and the facultative filter feeder *Protoglossus graveolens* and is higher in the gills of the deposit feeder *Saccoglossus bromophenolosus*. This finding supports the idea that as the functional selection is relaxed, developmental noise augments. *S. bromophenolosus* has significantly fewer gill slit than the other species, but they have not completely disappeared, and therefore may be regarded as a trait that exhibits persistence, where there is no cost in maintaining the trait, rather than vestigial. Further evidence for persistence is that gills are found in all eighteen‐described species of *Saccoglossu*s (Cameron et al., 2010). Another explanation for the persistence of the gills may be that they serve to eliminate water that is squeezed from sediment collected via deposit feeding (Burdon‐Jones, 1962; Gonzalez & Cameron, 2009). The collagenous bars may provide some skeletal support to these frangible worms or keep the pharynx from collapsing in on itself. We refrain from comparing the functions of the cephalochordate gills because the animals reside in a different environment, the larva exhibits a left‐handed directional asymmetry, and they do not deposit feed.

We found that the gills of *S. bromophenolosus* and *P. graveolens* show fluctuating asymmetry in the number of gill slits in the pharynx without any side bias. These results were not consistent with the expression pattern of *Nodal* and *Pitx* genes in deuterostomes (Wlizla, 2011). The *Nodal* and *Pitx* pathway that control left‐right asymmetries in Chordata is expressed on the left side of the body (Boorman & Shimeld, 2002; Yu et al., 2002). In echinoderm larvae, both genes are expressed on the right side and they control the asymmetrical growth of the adult rudiment (Duboc et al., 2005; Luo & Su, 2012). In Hemichordata, *Nodal* and *Pitx* are expressed either symmetrically or on the right side and their role in controlling asymmetries is not clearly established (Röttinger et al., 2015; Wlizla, 2011). Asymmetries in hemichordates are restricted to the gonad of the pterobranch *Rhabdopleura* (Satoh & Holland, 2008), the proboscis pore (Grande et al., 2015; Röttinger et al., 2015), and the coiling direction of *Saccoglossus* (Wlizla, 2011). The single gonad of *Rhabdopleura* is an antisymmetry that is likely related to packaging a large egg inside of a small zooid. An asymmetrical expression of *Nodal* may be linked to the left position of the proboscis pore (Röttinger et al., 2015; Wlizla, 2011). This pore is on the left of most hemichordate species, but may be located on the midline, on the right, and in the acorn worm *Stereobalanus* it is paired and symmetrical (Cameron et al., 2010; Cameron & Ostiguy, 2013; Deland et al., 2010).

We found that the gills of the adult cephalochordate *B. floridae* exhibit FA, with a significant difference between left and right in favor of the left side. This adult stage left‐side bias was likely inherited from the larval stage. *Branchiostoma* larvae exhibit a left‐handed directional asymmetry that is mostly lost in the adult stage. The larval gills develop as a single row of gill slits, the future left slits, on the right side of the body (Boorman & Shimeld, 2002; Holland & Onai, 2012; Lankester & Willey, 1890; Willey, 1891). A second row of gills then develops above in an inferior number. While the second row assumes some maturity, the first row begins to migrate to the left side and gills begin to atrophy. When the migration of the first row of slit has arrived on the left side of the animal, Willey (1891) states that the numbers of left to right gills are equal. Our results do not reject this idea, but instead demonstrate that within a population of *B. floridae*, the left‐sided bias is maintained in some individuals. The gonads of the cephalochordate genera *Epigonichthys* and *Asymmetron* are only on the right side (Nishikawa, 2004), and the gill asymmetry of larval *Asymmetron* is less conspicuous than *Branchiostoma* (Holland & Holland, 2010; Igawa et al., 2017). Cephalochordates gills are presumed to become symmetrical at metamorphosis, but they maintain a trace of left‐sided asymmetry as adults in the neural innervation of the pharyngeal complex, in the offset between opposing gills (in *Branchiostoma lanceolatum*) and now in the bias of their FA (Jefferies, 2001; Willey, 1891, 1894). This study underlines the importance of quantifying asymmetrical variation in a population to detect a subtle left‐sided asymmetry bias in adult cephalochordates.

The development of the secondary gill bars of *Protoglossus graveolens* with respect to the primary gill bars was highly variable demonstrating heterochrony. The posterior most gills of acorn worms are the youngest and exhibit early stages of development. Initially, they are simple pores that then elongate into a slit by the downward extension of the primary gill bars. This gill slit is then divided into two by the downward extension of the secondary gill bar. The number of primary gill slits lacking secondary gill bars in *B. floridae* and *S. bromophenolosus* was invariably zero, one, or two, whereas those of *P. graveolens* had an average of 17 ± 5 and up to 29 primary gill slits. This delay in the timing of development of secondary gill bars, with respect to primary gill bars, is an example of heterochrony where the development of secondary gill bars was delayed in *P. graveolens* with respect to the ancestral state presumed to be still present in *S. bromophenolosus* and *B. floridae*. Functionally, *P. graveolens* is both a filter feeder (like *Branchiostoma*) and a deposit feeder (like *Saccoglossus*) so we found no relationship between the gill bars heterochrony, gill function, and fluctuating asymmetry.

We discovered one *S. bromophenolosus* specimen with gills at an earlier stage of development than the gills just posterior to them, suggesting regeneration following damage (Figure 6e). More surprising was the finding of an entirely new second pharyngeal complex in 12.3% of the *S. bromophenolosus* specimens. The developmental mechanism for this was likely an ectopic expression of pharyngeal developmental genes, perhaps induced from a population of cells that were passed posteriorly through the gut. This is a previously unknown form of regeneration for acorn worms, though regeneration in the group is well document. Acorn worms are susceptible to breakage, and the posterior fragments of experimentally bisected animals will regenerate new individuals, including a new pharynx, from both anterior and posterior fragments (Dawydoff, 1909; Humphreys et al., 2010; Rao, 1955; Rychel & Swalla, 2008, 2009; Tweedell, 1961; Willey, 1899; Miyamoto & Saito, 2010). A duplicate pharynx complex was found in one *P. graveolens,* and none were found in the *B. floridae* specimens. Like the higher FA, a second pharynx complex buttresses the hypothesis that the gills of the deposit feeder *S. bromophenolosus* are more prone to developmental error than those of the filter feeders. Drift may predominate over selection in *Saccoglossus* gill development, though adaptive hypotheses cannot be ruled out. A second set of collagenous gill bars, for example, may resist breakage of the trunk or maintain an open gut that in turn facilitates the passage of sediment or the absorption of nutrients. The instability seen in *S. bromophenolosus* gill slits is not reflected in the only other asymmetrical structure of Enteropneusta, the proboscis pore. Some enteropneusts are known to have the pore on one side or the other (Deland et al., 2010), but none from the genus *Saccoglossus* (Cameron et al., 2010).

The hypothesis that gill used in filter feeding evolved before the divergence of the hemichordate‐echinoderm clade from the chordates (Cameron, 2002) has gained support from comparative morphology (Gonzalez & Cameron, 2009), molecular development (Lowe et al., 2003; Ogasawara et al., 1999; Okai et al., 2000), comparative genomics (Simakov et al., 2015), biomechanics (Vo et al., 2019), and the discovery of Cambrian acorn worm fossils (Caron et al., 2013; Nanglu et al., 2016). Here, we append to this hypothesis that the last common ancestor of Hemichordata and Cephalochordata was bilaterally symmetrical. Our finding is limited by the number of species analyzed, but in the context of the hypothesis above, our results provide a robust tendency within the study groups and bring evidence that gills of species that have experienced a relaxation of the filter feeding trait exhibit elevated FA. This finding is significant because it rejects hypotheses that the deuterostome ancestor was asymmetrical (Jefferies et al., 1996; Sato & Holland, 2008). Instead, the directional asymmetry of stem group fossil echinoderm (Smith, 2008; Zamora & Rahman, 2014; Zamora et al., 2012) and cephalochordates evolved independently (Igawa et al., 2017; Kaji et al., 2016). This study also cautious the use of single specimens to determine symmetry of nearly symmetrical fossil or extant body plans. A 3D‐reconstruction of a juvenile *Saccoglossus kowalevskii* found left side first asymmetrical development of the gills (Kaul‐Strehlow & Stach, 2013). We interpret this small asymmetry as a spandrel of the preferential right‐handed coiling in prehatching embryos, rather than as an indication of directional or antisymmetry (Wlizla, 2011). The symmetry of a species cannot be determined from an individual specimen because populations of bilateral species are comprised of individuals that exhibit variations around that mean, or FA, due to developmental noise. The deposit feeder *Saccoglossus* exhibits the highest noise, demonstrating that a loss in filter function relaxes selection on this trait. Likewise, pterobranchs use tentacles to filter‐feed instead of the gills which are vestigial or lost in extant pterobranchs (Dilly, 1985; Lester, 1985; Vo et al., 2019). The enteropneust family Torquaratoridae is a family of deep‐sea deposit feeder who also show signs of vestigialization of their gills (Holland et al., 2009; Jabr et al., 2018). Filter feeding, then, appears to be the original selective role of invertebrate deuterostome gills, rather than the sorting and transport of sediment (Knight‐Jones, 1953) and respiration, and excretion may have been of little importance.

## Conflict of interest

All authors declare no conflict of interest and that all experiments were done in an ethical manner.

## Author contribution

**Charles Larouche‐Bilodeau:** Data curation (equal); Formal analysis (equal); Investigation (lead); Methodology (supporting); Project administration (supporting); Resources (supporting); Software (supporting); Visualization (equal); Writing‐original draft (lead); Writing‐review & editing (lead). **Xavier Guilbeault Mayers:** Conceptualization (equal); Data curation (lead); Formal analysis (equal); Investigation (equal); Methodology (equal); Project administration (supporting); Software (lead); Visualization (lead); Writing‐original draft (supporting); Writing‐review & editing (supporting). **Christopher B. Cameron:** Conceptualization (lead); Data curation (supporting); Formal analysis (supporting); Funding acquisition (lead); Investigation (supporting); Methodology (supporting); Project administration (equal); Resources (lead); Supervision (lead); Validation (lead); Writing‐original draft (equal); Writing‐review & editing (supporting).

## Data Availability

The code and data used for the statistical analysis has been deposited in Dryad and is accessible at https://doi.org/10.5061/dryad.ksn02v72r. The code used to perform the Student's *t* test by permutation for paired sample is available at http://adn.biol.umontreal.ca/~numericalecology/Rcode/.
